# Clinical use of lithium salts: guide for users and prescribers

**DOI:** 10.1186/s40345-019-0151-2

**Published:** 2019-07-22

**Authors:** Leonardo Tondo, Martin Alda, Michael Bauer, Veerle Bergink, Paul Grof, Tomas Hajek, Ute Lewitka, Rasmus W. Licht, Mirko Manchia, Bruno Müller-Oerlinghausen, René E. Nielsen, Marylou Selo, Christian Simhandl, Ross J. Baldessarini, for the International Group for Studies of Lithium (IGSLi)

**Affiliations:** 1 International Consortium for Research on Mood & Psychotic Disorders, McLean Hospital, Belmont, MA USA; 2 Department of Psychiatry, Harvard Medical School, Boston, MA USA; 3 Lucio Bini Mood Disorders Centers, Lucio Bini Center, Via Cavalcanti 28, 09128 Cagliari and Rome, Italy; 4 Department of Psychiatry, Dalhousie University, Halifax, NS Canada; 5 Department of Psychiatry and Psychotherapy, Carl Gustav Carus University Hospital Dresden, Dresden, Germany; 6 Department of Psychiatry and Department of Obstetrics, Gynecology and Reproductive Science, Icahn School of Medicine at Mount Sinai, New York, USA; 7 Department of Psychiatry, Erasmus Medical Center, Rotterdam, The Netherlands; 8 Mood Disorders Center of Ottawa and Department of Psychiatry, University of Toronto, Toronto, Canada; 9 Department of Clinical Medicine, Aalborg University, Aalborg, Denmark; 10 Aalborg University Hospital–Psychiatry, Aalborg, Denmark; 11 Department of Pharmacology, Dalhousie University, Halifax, NS Canada; 12 Section of Psychiatry, Department of Medical Sciences and Public Health, University of Cagliari, Cagliari, Italy; 13 Drug Commission of the German Medical Association, Berlin, Germany; 14 International Advocacy, New York, USA; 15 Medical Faculty, Bipolar Center, Sigmund Freud Private University, Wiener Neustadt, Austria

**Keywords:** Bipolar disorder, Blood testing, Dosing, Lithium, Side-effects

## Abstract

**Background:**

Lithium has been used clinically for 70 years, mainly to treat bipolar disorder. Competing treatments and exaggerated impressions about complexity and risks of lithium treatment have led to its declining use in some countries, encouraging this update about its safe clinical use. We conducted a nonsystematic review of recent research reports and developed consensus among international experts on the use of lithium to treat major mood disorders, aiming for a simple but authoritative guide for patients and prescribers.

**Main text:**

We summarized recommendations concerning safe clinical use of lithium salts to treat major mood disorders, including indications, dosing, clinical monitoring, adverse effects and use in specific circumstances including during pregnancy and for the elderly.

**Conclusions:**

Lithium continues as the standard and most extensively evaluated treatment for bipolar disorder, especially for long-term prophylaxis.

## Historical introduction

In 1949, John Cade (1929–1996), an Australian psychiatrist, serendipitously initiated a new era in psychiatric treatment by using lithium carbonate to treat mania. His use of lithium arose from the hypothesis that major mental illnesses might be associated with deficiencies or excesses of unidentified chemical substances, including accumulations of nitrogenous metabolites. This idea led him to give lithium carbonate to laboratory animals to limit toxicity of test substances including uric acid and noting calming and other behavioral changes. Subsequently, he reported on beneficial effects of treating ten patients with lithium carbonate (a medically accepted, though unproved, treatment for gout) for mania and on risks of discontinuing such treatment (Cade 1949). These encouraging initial results are now widely considered a revolutionary discovery, although this innovative and effective treatment was not immediately adopted by psychiatry. As Cade himself observed, “a discovery by an unknown psychiatrist without research training, working in a small hospital for the chronically mentally ill, with primitive techniques and negligible equipment, could not attract much attention” (Cade 1999). In addition, several cases of severe, acute intoxication associated with use of lithium salts as a substitute for table salt (sodium chloride) were reported in 1949, and some experience was required to learn how to use lithium safely. This could be achieved by measuring its concentration in blood (Amdisen 1967; Baldessarini 2013; Bauer and Gitlin 2016).

In 1954, Professor Erik Strömgren (1909–1993), a prominent academic psychiatrist at the University of Aarhus Medical Center in Denmark, read of Cade’s experience with lithium for mania and asked his then-junior colleague Mogens Schou (1918–2005) to replicate the Australian findings. Schou not only did so but pursued the study of lithium to contribute in a major way to establishing its safe and effective clinical use for the treatment of manic-depressive illness. His research in collaboration with another Danish psychiatrist, Poul Baastrup (1918–2001), established the most important effect of lithium—its ability to prevent recurrences of manic-depressive illness (Baastrup 1964; Baastrup and Schou 1968). In Zurich, Jules Angst (born 1926) and his collaborators confirmed the prophylactic benefits of lithium treatment (Angst et al. 1970). In addition, Angst with Paul Grof (born 1935) and other colleagues proposed methods of clinical trial-design suitable for testing for long-term, prophylactic effects of a treatment such as lithium (Grof et al. 1970).

Gradually lithium became accepted in clinical practice around the world, though not without some resistance. There was, in fact, a strong skepticism about its long-term efficacy, based on reasonable considerations (Blackwell and Shepherd 1968). These included the requirement for evidence from blinded, randomized, controlled trials. The first of these helped to convince the scientific and clinical community (Baastrup et al. 1970). Lithium treatment finally received regulatory approval by the US Food and Drug Administration (FDA) in 1970 for treatment of acute mania, and in 1974 as the first—and for many years, the only—approved treatment for prevention of recurrences in bipolar disorder. In addition to its potentially toxic effects, lithium being an unpatentable natural product with limited commercial interest had difficulties in attracting extensive support for research or clinical promotion by pharmaceutical corporations.

Clinical applications of lithium were made feasible by introducing sensitive, reliable, quantitative methods of monitoring lithium concentrations in serum, initially with flame spectrophotometry and later with atomic absorption, electrochemical, and other detection methods. These established circulating concentrations of lithium required for safe and effective clinical dosing (Amdisen 1967; Bauer and Gitlin 2016). Such monitoring was required because of the narrow margin between the safe and potentially toxic dose of lithium, or its “therapeutic index” (ratio of median toxic to therapeutically effective doses, of approximately 3) (Baldessarini 2013; Bauer and Gitlin 2016; Perugi et al. 2019). Indeed, lithium remains unique in not being dosed adequately by the mg dose of drug given per day, but instead by achieving serum concentrations 10–14 h after the last taken dose (most stable range of the day) on the range of 0.5–1.0 mEq/L, while being aware that earlier, daily peak concentrations can be 2–3-times higher (Amdisen 1967; Baldessarini 2013; Bauer and Gitlin 2016; Perugi et al. 2019).

## Lithium today

Lithium treatment remains the “gold standard” of treatment for preventing recurrences in bipolar disorder, both types I (with mania and major depression) and II (with depression and hypomania). It also has evidence of effectiveness for preventing suicidal behavior in patients with bipolar or major depressive disorder. However, it has gradually become less widely utilized, particularly for mania, mainly due to more vigorously promoted and more rapidly effective alternatives which do not require blood tests. These alternatives include drugs developed for other purposes, including certain anti-epilepsy agents (carbamazepine, lamotrigine, sodium valproate) and most antipsychotics. Their adverse effects include metabolic syndrome (weight-gain with diabetes, high blood pressure, and increased lipids in the blood), insulin resistance, abnormal movements, and cognitive dulling with antipsychotics, as well as markedly increased risks of birth defects including spina bifida and severe cardiac anomalies during pregnancy in association with valproate and carbamazepine (Patel et al. 2018). Concerns about the safety of lithium have not entirely disappeared, despite long-established standards for its safe use with monitoring of its serum concentrations.

Fears about lithium treatment currently are most often directed to putative renal toxicity with its long-term use. In fact, such effects are uncommon and usually can be anticipated by rising serum concentrations of creatinine or declining creatinine clearance (McKnight et al. 2012; Baldessarini 2013; Bauer and Gitlin 2016; Haussmann et al. 2017; Perugi et al. 2019). This adverse effect of lithium is modest, and greatly overlaps with age-associated declining renal function (Nielsen et al. 2018). An indication of declining renal function (at least one elevated serum concentration of creatinine) was documented in about 30% of subjects treated with lithium for 15 years or more and aged 55 years or older (Tondo et al. 2017).

Some patients and families may feel that lithium treatment has a “stigmatizing” effect, as lithium is perceived as a medication for severely ill patients, in contrast to seemingly less stigmatizing anticonvulsant, antidepressant, antipsychotic and other psychotropic medicines (Baldessarini 2013; Bauer and Gitlin 2016). In some countries, particularly in the US, vigorous promotion of alternative and patented treatments, sometimes more rapidly acting against mania, have led to substantial displacement of lithium for acute mania. Nevertheless, lithium continues to hold a major position among treatments for bipolar disorder internationally, especially for long-term prophylactic treatment, and it tends to be used for longer times than most alternatives (Baldessarini et al. 2007, 2008, 2019b).

## Efficacy of lithium treatment

Although lithium is effective in treating acute manic episodes, its primary value is as a “mood-stabilizing” agent, aiming at long-term prevention of recurrences of acute illness-episodes in bipolar disorder patients (Baldessarini 2013; Bauer and Gitlin 2016; Haussmann et al. 2017; Malhi et al. 2017; Perugi et al. 2019), with greater benefit against recurrences of mania than for bipolar depression (Kleindienst and Greil 2000), as the case also with alternative treatments (Baldessarini 2013; Forte et al. 2015). Satisfactory mood-stabilization over 6–12 months can be attained by about two-thirds of lithium-treated bipolar disorder patients, and an excellent response (e.g., 3 years with no recurrences) has been found in one-third of such patients (Grof 2006). Recent comprehensive reviews considered the benefits of various proposed mood-stabilizing treatments, including lithium, against recurrences of mania and depression in bipolar disorder patients, in more than a dozen placebo-controlled, randomized, long-term trials carried out for an average of 1.5 years (Geddes et al. 2004; Popovic et al. 2011, 2012: Vieta et al. 2011; Miura et al. 2014). Such studies found that long-term risk of new manic or depressive episodes was lower with lithium than with placebo, although the benefit was greater against new episodes of mania than of depression. Indeed virtually all available treatments for bipolar disorder, including lithium, anticonvulsants, and antipsychotics are notably limited in their effectiveness against recurrences or acute episodes of bipolar depression and their long-term recurrences, with the exception of lamotrigine for long-term treatment (Geddes et al. 2004, 2010; Poon et al. 2012; Baldessarini 2013; Vázquez et al. 2013; Forte et al. 2015). However, some randomized, double-blind trials from the 1970s and several recent studies found that long-term treatment with lithium also may reduce recurrences in unipolar major depressive disorder (Abou-Saleh et al. 2017; Tiihonen et al. 2017; Undurraga et al. 2019). In addition, lithium appears to have value in augmenting antidepressant treatment, especially during episodes of unipolar major depression that respond unsatisfactorily to antidepressant treatment. Most studies supporting this application have involved older, tricyclic antidepressants, but similar effects may occur with modern antidepressants as well (Austin et al. 1991; Bauer and Döpfmer 1999; Bauer et al. 2003; Alevizos et al. 2012; Bauer and Gitlin 2016; Undurraga et al. 2019). Finally, it has been suggested that lithium occurring naturally in drinking water may lower the incidence of dementia, but this finding needs to be verified (Kessing et al. 2017a, b).

## Differential response to lithium

Mood-stabilizing and prophylactic benefits of lithium may be particularly evident in patients with typical bipolar disorder that includes an episodic clinical course before treatment, a family history of the disorder and favorable response by a family member, lack of other co-occurring psychiatric illnesses, and an illness course-sequence characterized by mania or hypomania followed by depression and then a stable or euthymic interval (“MDI” pattern) rather than the opposite (depression-mania-euthymic interval (“DMI”)) (Koukopoulos et al. 2013; Malhi et al. 2017; Yatham et al. 2018). In particular, patients with an MDI course-type (especially likely in type I bipolar disorder) have shown a 29% (CI 18–40%) better response to lithium than those with a DMI course (more likely in type II bipolar disorder and often triggered by overuse of antidepressants) (Kukopulos et al. 1980; Koukopoulos et al. 2013). This association with the type of illness-course is consistent with the view that depression-prone bipolar disorder patients, generally, respond less favorably to treatment than mania-prone patients (Vieta et al. 2009; Baldessarini et al. 2012). Of interest, both the DMI versus MDI recurrence pattern, as well as the tendency to a long-term excess of mania over bipolar depression may be identifiable very early on the illness history, possibly from the first-lifetime episode or cycle (Baldessarini et al. 2012; Koukopoulos et al. 2013; Forte et al. 2015).

Responsiveness to lithium (and alternative treatments) has been inferior among patients with relatively complicated forms of bipolar disorder, such as with rapid-cycling, psychotic features, co-occurring anxiety syndromes or substance-abuse, as well as in depression-prone cases or those following the DMI course pattern, and lithium is less effective in preventing recurrences of bipolar depression than mania. Importantly, the same limitations hold true for alternative treatments, including anticonvulsants and modern antipsychotics (Baldessarini et al. 2002; Tondo et al. 2003; Bauer and Gitlin 2016), and no alternative treatment appears consistently to outperform lithium for long-term maintenance treatment (Geddes et al. 2004, 2010; Baldessarini 2013; Pacchiarotti et al. 2013; Vázquez et al. 2013; Bauer and Gitlin 2016; Malhi et al. 2017; Yatham et al. 2018). Moreover, as for all maintenance treatments, a favorable clinical response is associated with faithful adherence to the treatment regardless of current lack of symptoms, absence of early or current adverse life events, adult age at onset, good social support, and absence of substance abuse or other co-occurring psychiatric disorders including personality disorders (Yatham et al. 2018).

## Starting and discontinuing treatment and medical monitoring

Whether indefinitely continued maintenance treatment should be started routinely after an initial manic episode lacks clear consensus. A conservative view would initiate long-term treatment after a second episode of mania since the first might be followed by another only after several years or may be of only moderate severity and duration. Typically, patients are continued on lithium or an alternative treatment for at least some months following recovery from an acute episode of mania or bipolar depression, with reassessment of the need to continue thereafter. Lithium probably should be started after a first manic episode presenting with severe symptoms, required hospitalization, or involved suicidal risk or prolonged duration. In general, early long-term intervention and follow-up are encouraged after a manic episode, and especially of juvenile onset, because of the adverse impact of bipolar illness on a patient’s educational, occupational, and social functioning (Kessing et al. 2017a, b).

Because of the frequent lack of timely recognition and diagnosis of bipolar disorder, especially of type II, initiation of long-term treatment with lithium or other mood-stabilizing treatments typically does not occur for 5–10 years from illness-onset, and even longer following juvenile onset (Post et al. 2010; Kessing et al. 2017a, b). Such delay, with associated morbidity, contribute considerably to disability and risk of suicide. Nevertheless, some studies have found that neither such delays nor the number of recurrences before treatment had a measurable impact on the likelihood of responding once mood-stabilizing treatment was initiated (Baethge et al. 2003; Bratti et al. 2003; Berghöfer et al. 2008).

Before starting treatment with lithium, and once or twice a year thereafter, we recommend measuring blood concentrations of (a) creatinine and urea-nitrogen (BUN) to evaluate kidney function, (b) sodium, potassium, calcium, and (c) thyroid and parathyroid hormones, as well as obtaining an electrocardiogram—all of which can be affected by lithium.

Discontinuing long-term lithium treatment of mood disorder patients leads to a substantial risk of illness-recurrences, need for hospitalization, and of suicidal behavior, especially if the discontinuation is rapid or abrupt. The risk is especially high in the 6–12 months after discontinuation (Faedda et al. 1993; Baldessarini and Tondo 2019). Of note, recurrences of bipolar disorder following discontinuation of maintenance treatment may be more severe and occur much sooner when lithium is discontinued rapidly (< 15 days) or abruptly (Faedda et al. 1993; Baldessarini 2013). This effect not only reflects lack of treatment but may be a response to treatment-discontinuation itself as a major stressor (Baldessarini and Tondo 2019).

## Lithium and suicide

Patients with bipolar disorder have an increased standardized mortality ratio (SMR) for all-cause mortality as compared to the general population, with higher rates of suicide at young ages and of fatal medical illnesses in late years (Ösby et al. 2001; Staudt-Hansen et al. 2019). SMR for suicide as the cause of death is far higher with bipolar disorder and severe, recurrent, non-bipolar major depression (especially involving psychiatric hospitalization) than other psychiatric disorders, up to 20-times higher than the international general population rate of approximately 15/100,000/year (0.015%/year) (Harris and Barraclough 1997; Ösby et al. 2001; Simon and Hales 2012; Baldessarini et al. 2019a, b, c). This excess of mortality is strongly associated with suicide in younger patients, but with co-occurring medical disorders in older patients (Ösby et al. 2001; Baldessarini et al. 2019a). Suicide is far more likely in depressive, and especially dysphoric-agitated or mixed, phases of bipolar disorder than in manic periods, and is rare in hypomania (Swann et al. 2013; Tondo and Baldessarini 2016). The rate of suicide attempts is at least 20–30 times greater than rates of suicide in the general population but the ratio of suicides/attempts is much lower among patients with a major mood disorder (≤ 10). This ratio indicates greater lethality of means and intent in major mood disorder patients with a similar risk of suicidal behavior in types I and II bipolar disorder (Tondo et al. 2016).

Initial considerations that lithium treatment might contribute to suicide prevention date to the early 1970s (Barraclough 1972), followed by important contributions supporting this hypothesis over the next two decades (Coppen et al. 1991; Coppen and Farmer 1998). An early case–control study of suicidal risk in 68 patients with various major affective disorder diagnoses and at least one suicide attempt, found a rate of suicides or attempts during 8.0 years of lithium treatment of 1.1%/year, with a highly significant increase to 2.0%/year following discontinuation of lithium treatment (Müller-Oerlinghausen et al. 1992). In 360 type I or II bipolar disorder patients before, during, and following discontinuation of long-term lithium monotherapy, rates of suicide and life-threatening attempts were 6.4-times lower during lithium-treatment than either before (only attempts) or long after treatment. The risk of suicidal acts increased 20-fold within several months after discontinuing lithium maintenance treatment, but later fell back to the same level encountered before lithium treatment had started (Tondo et al. 1998). Moreover, early suicidal risk following discontinuation of long-term treatment with lithium was twice-higher following abrupt or rapid versus more gradual discontinuation of lithium over at least 2-weeks.

In a systematic review of 12 studies, the pooled rate of suicides and attempts was 8.9-times lower with lithium treatment (*p *< 0.0001) (Tondo et al. 2001). In addition, a review of 8 studies on unipolar recurrent major depression patients found that long-term lithium treatment was again associated with a substantial reduction of risk of suicides and attempts (by approximately 76%) among patients treated with lithium compared to other alternatives, mainly antidepressants and anticonvulsants (Guzzetta et al. 2007). Similar effects on suicidal behavior have not been achieved with several proved or putative mood-stabilizing anticonvulsants (including carbamazepine, lamotrigine, and valproate) (Thies-Flechtner et al. 1996; Goodwin et al. 2003; Baldessarini et al. 2009; Smith et al. 2009). Antipsychotic drugs have not been tested for such effects in bipolar disorder patients. An association of reduced suicidal risk during long-term treatment with lithium in bipolar disorder patients is supported by several studies and quantitative reviews (Angst et al. 2005; Cipriani et al. 2005, 2013; Kessing et al. 2005; Müller-Oerlinghausen et al. 2006; Lauterbach et al. 2008; Lewitzka et al. 2013). It may be that an antisuicidal effect of lithium treatment is at least partly independent from its mood-stabilizing effect (Ahrens and Müller-Oerlinghausen 2001; Manchia et al. 2013). If so, patients achieving only a partial clinical response to lithium treatment might still benefit from its antisuicidal effects (Ahrens and Müller-Oerlinghausen 2001).

Overall, the findings summarized here, appear to provide strong and quite consistent support for the hypothesis that lithium treatment may have a special role in reducing suicidal risks. Although these findings are encouraging and unusual, it must be emphasized that such an effect of lithium treatment has not yet been definitely proved in prospective, randomized trials in which suicidal behavior is an explicit outcome. Largely for that reason, lithium has not been accepted by the FDA as a specific indication to prevent suicidal ideation or behavior. Such an indication was requested in a citizen’s petition to FDA by Drs. Frederick Goodwin and Ross J. Baldessarini. This included data from several randomized, controlled trials in which suicidal acts were cited as adverse outcomes. However, no pharmaceutical company is likely to underwrite studies or to make formal application to the FDA for such an indication. The only randomized and controlled study in patients who have attempted suicide, with suicidal behavior as an explicit outcome, found a significantly lower number of suicides in the lithium group compared to placebo (0 vs. 3 suicides; *p *= 0.049) but not in the number of suicide attempts (7 vs. 10 attempts) (Lauterbach et al. 2008).

## Lithium dosage and blood concentration

The following guidelines for managing lithium treatment are based on several recent publications and the extensive clinical experience of the authors (Baldessarini 2013; Bauer and Gitlin 2016; Haussmann et al. 2017; Malhi et al. 2017; Perugi et al. 2019). As noted, lithium is used mostly as a long-term treatment to prevent mood-disorder recurrences. Its place in the treatment of acute mania has largely been displaced in favor of some anticonvulsants and modern antipsychotic drugs, which act more rapidly and whose target doses can be reached within a few days. In particular, the most common current treatment for a manic episode is with modern antipsychotic agents for several months, with lithium introduced adjunctively or continued long-term by itself as a preventive treatment (Yatham et al. 2018).

Lithium should be taken regularly as prescribed. A daily single dose after the evening meal is convenient, preferably with slow-release formulations in relatively young, otherwise healthy patients. This practice can support critically important, long-term treatment-adherence (Malhi et al. 2017). For older or infirm patients, and users of high daily doses (over 1200 mg of lithium carbonate [32 mEq]), divided daily doses may be safer. If a dose is missed it is not safe to double the next dose. If the brand or salt-form of lithium needs changing, this should be done by gradually discontinuing the first preparation as the second is introduced and gradually increased. The optimum amount of lithium to be taken is based on clinical response and measured blood levels of lithium which guide the dose of lithium. Blood assays of lithium usually are obtained at 1 week after the start of lithium treatment, then monthly in the first 3 months. Subsequently, when the patient is considered stable, blood tests may be done every 6 months depending on age, general health, and response to treatment. Blood should be drawn at a consistent interval, at 10–14 (optimally, 12) hours after the last intake. If a dose is changed, 5–7 days should pass before measuring the blood level to allow tissue distribution to stabilize. Optimal doses of lithium are decided by a clinician, and depend on the patient’s age, general health, type of bipolar disorder, symptom-severity, and frequency of recurrences. Optimal daily trough blood concentrations of lithium for long-term treatment usually are between 0.50 and 0.60 and 0.80–1.00 mEq/L. Some patients may require higher concentrations, whereas for others, lower concentrations may suffice and better tolerated. For example, higher serum levels of lithium may be required for young patients with severe manic or psychotic symptoms (delusions, hallucinations) or patients with short intervals between episodes, whereas lower concentrations are often used and better tolerated by elderly patients.

Lithium dose is best decreased by half or held for some days in cases with fever above 38 °C (100.4 °F), dehydration or diarrhea. Lithium also should be taken with extra caution during treatment with certain medicines that can slow its renal elimination and increase risk of toxic effects, and if a low-sodium diet is required for medical reasons. Such drugs include the commonly used nonsteroidal anti-inflammatory drugs (NSAIDs) such as ibuprofen or nimesulide if they need to be taken more than occasionally. Lithium blood level measurements are recommended if such drugs are taken regularly for extended periods. However, a safer treatment for pain or fever than an NSAID is acetaminophen/paracetamol. Certain anti-hypertensives (notably angiotensin-converting enzyme [ACE] inhibitors), some drugs used to treat cardiac arrhythmias, and most diuretics should be used with caution. During use of lithium with any of these medicines, serum lithium levels may rise and should be monitored frequently. In addition, lithium treatment should be stopped 48–72 h before surgery that requires general anesthesia, and while a patient does not drink or eat as usual. For surgeries requiring local anesthesia, no interruption is needed. Lithium may be resumed when the patient is well hydrated (Huyse et al. 2006). Finally, lithium should be stopped during electroconvulsive treatment (ECT) as a precaution to prevent possible neurological symptoms (delirium) (Tsujii et al. 2019).

## Adverse effects and contraindications

Some adverse effects are common during treatment with lithium. These include tremor (a dose-dependent effect, which can be treated with low doses of the centrally active, beta-adrenergic blocker propranolol, high doses of vitamin B6, or with dose reduction if possible), nausea, fatigue, increased appetite, increased white blood-cell count, thirst, and increased frequency of urination (polyuria). This symptom may respond to cautiously added, small doses of the diuretic hydrochlorothiazide (which can also increase serum concentrations of lithium and decrease potassium). Some patients complain of decreased cognitive functions. Some of these adverse effects (especially thirst and tremor) tend to disappear over the first weeks of treatment. Gastro-intestinal complaints may be lessened by switching to another lithium preparation. Hypothyroidism is also possible and usually is treated with supplemental thyroid-hormone (Ambrosiani et al. 2018; Bocchetta et al. 2018). Hyperparathyroidism and consequent hypercalcemia can also arise during long-term treatment with lithium (Twigt et al. 2013).

Lithium should not be used by patients who have or have had: acute myocardial infarction, acute kidney failure, or certain rare disorders of heart rhythm (notably, the Brugada syndrome of ventricular arrhythmia). Lithium can be used cautiously and with close medical monitoring in the presence of: cardiac arrhythmia, reduced kidney function, psoriasis, myeloid leukemia, Addison’s disease, hypothyroidism, and certain neurological disorders, including abnormalities of posture and movement, myasthenia gravis, and epilepsy. A rare, probably irreversible, syndrome acronymized as SILENT (Syndrome of Irreversible Lithium-Effectuated Neurotoxicity) involving persisting cerebellar dysfunction with ataxia or unsteady gait has been reported in a few cases (Aditanjee et al. 2005).

In older patients, doses of lithium should be about 20% lower than those for younger patients, as determined by the attending clinician (Shulman et al. 2019). Common adverse effects in the elderly, in addition to those already described, can include: confusion or worsening of cognitive functions, unsteady balance and gait (ataxia), restless movements (akathisia), declining kidney function, hypothyroidism, possible worsening of diabetes mellitus, and leg-swelling (peripheral edema).

Abnormally high blood levels of lithium (above 1.5 mEq/L) can increase risk of intoxication, signs of which are severe tremor, confusion, vomiting, abdominal pain, diarrhea, abnormally increased reflexes, speech difficulties, abnormal heart rhythm, hypotension, and convulsions. The prescribing clinician or an emergency service should be consulted immediately if such symptoms occur. Blood tests to determine the level of lithium is required. If it is very high (above 2 mEq/L), blood dialysis may be needed to remove lithium—a decision best made in consultation with a kidney specialist. Even if measured blood levels lithium are not greatly elevated, intoxication may be present due to circulating levels in blood not being in equilibrium with those in the central nervous system, and lithium should be stopped as clinical evaluation is pursued.

## Lithium in pregnancy and post-partum

Use of lithium in pregnancy has long been a matter of discussion. In the past, the mainstream opinion was that lithium should be discontinued immediately when pregnancy was ascertained or planned. The major concern was risk of a rare congenital cardiac abnormality (Ebstein’s anomaly, arising in approximately 1 in 20,000 live births without lithium exposure). Recent studies have confirmed the association between maternal exposure to lithium in the first 3 months of pregnancy and increased risk of this and other fetal malformations but their risk-estimates are lower than previously reported (Patorno et al. 2017; Munk-Olsen et al. 2018; Poels et al. 2018). Reduction of lithium doses during the first trimester may be considered but weighed against the especially high risks of relapse (especially of depressive or mixed states of bipolar disorder) early in pregnancy associated with treatment discontinuation (Viguera et al. 2007). When lithium is prescribed during pregnancy, serum concentrations should be monitored frequently (at least monthly), and preferably weekly in the third trimester (Wesseloo et al. 2017). It is not necessary to stop lithium before delivery, but blood levels should be measured twice weekly for the first 2 weeks after delivery (Wesseloo et al. 2017). If delivery is prolonged, it is vital to ensure a good fluid intake during treatment with lithium. Clinicians and patients should discuss risks and benefits of continuing, lowering, or interrupting lithium treatment in anticipation of pregnancy and after childbirth, including during breast-feeding (Poels et al. 2018).

If lithium is discontinued during pregnancy in a woman with bipolar disorder, it should be restarted immediately after delivery, because the early post-partum period carries a high risk for recurrences of bipolar disorder and depression (Viguera et al. 2007; Wesseloo et al. 2016). Serum lithium levels are often increased to 0.8–1.0 mEq/L during the first month after childbirth to minimize risk of illness and checked at least weekly for a month. If the blood level is stable, it can be checked monthly for the next 3–6 months. Breast-feeding during lithium treatment is not recommended (as concentrations in breast milk are about half of the concentrations in maternal blood), and infant feeding formula should be used (Galbally et al. 2018; Poels et al. 2018).

Whenever lithium is discontinued, this should be done gradually, over at least a month at a rate of dose-reduction of 20–25% every 2 weeks or longer. If an acute medical problem requires rapid discontinuation, this needs to be carried out under close medical supervision.

## Conclusions

In addition to publications cited above (Baldessarini 2013; Bauer and Gitlin 2016; Baldessarini et al. 2019a, b, c; Perugi et al. 2019), several helpful guidelines concerning bipolar disorder and its treatment have appeared in recent years (Licht et al. 2003; Goodwin et al. 2016; Grunze et al. 2013; Fountoulakis et al. 2017; Haussmann et al. 2017; Malhi et al. 2017; Nederlof et al. 2018; Yatham et al. 2018). These publications may not be readily available to many physicians working outside of large academic or clinical institutions. Moreover, many patients rely on information from the internet, which may be unreliable. These circumstances led us to prepare of this report, which aims to provide a brief and accessible but expert overview for patients, their caregivers (parents, spouses, children, siblings, or friends), and their doctors. Key points are summarized in Table 1, and address questions that patients are most likely to have about lithium treatment. These points and specific questions should be discussed with each patient’s personal clinician prescribing lithium.

**Table 1:** Recommendations for patients treated with lithium and their prescribers

*Background* Lithium salts (carbonate, citrate, or sulphate) have been widely used for about 70 years to treat illnesses characterized by periods of depression and elevated or excited mood (bipolar disorder) or with depression only (major depressive disorder). Lithium and other mood-stabilizing treatments do not cure mood disorders but can reduce the frequency, severity, and duration of relapses and improve long-term stability
*Indications* Lithium is especially effective in preventing recurrences of excited (manic) mood and can also reduce the risk of depressive recurrences. It is the only medicine with evidence of reducing risk of suicidal behavior associated with mood disorders. It may also reduce risk of cognitive decline in the elderly
*Dosing* Lithium should be taken regularly, exactly as prescribed. If a dose is missed, do not double the next dose. A single daily dose is sometimes used and more convenient than divided doses. Changing the brand or type of lithium salt used may require dosage re-adjustment with supervision by your doctor
*Blood concentrations of lithium* Measuring the blood level of lithium is very important. It should be done about 1 week after the first dose, then weekly in the first month, at least once a month in the next 3–6 months, and every 3–6 months thereafter. Blood sampling should be done reliably at a consistent interval (optimally about 12 h after the last dose of the day) and about a week after any dose change. Prescribed dosing is guided by blood concentrations of lithium decided by your doctor, usually at 0.50–0.80 mEq/L
*Factors that can alter blood levels* Talk with your doctor about lowering your daily dose of lithium or to discontinue temporarily with: fever above 38 °C (100.4 °F), dehydration or diarrhea, or when a low sodium diet is required (not recommended during lithium therapy). Long-term use of nonsteroidal anti-inflammatory drugs (NSAIDs) is not recommended with lithium (acetaminophen/paracetamol is preferred for pain). ACE-inhibitors, some cardiac antiarrhythmics, and thiazide-diuretics should not be used with lithium
*Other blood tests* Blood levels of creatinine (for kidney function), sodium, potassium, calcium, thyroid and parathyroid hormones should be measured before starting the treatment and at least, once or twice a year thereafter
*Adverse effects* Nausea, thirst, tremor, fatigue, decreased cognitive functions, increased appetite, increased frequency of urination. These are most often encountered early in treatment and usually improve with time, but may require additional treatments
*Use in medical conditions* Lithium should not be used by patients who have or have had: acute myocardial infarction (heart attack), acute kidney failure, or certain rare disorders of heart rhythm. It can be used cautiously and with close medical monitoring with: cardiac arrhythmia, reduced kidney function, psoriasis, myeloid leukemia, Addison’s disease, hypothyroidism, and certain neurological disorders, including abnormalities of posture and movement, tremors, myasthenia gravis, and epilepsy. Lithium should be stopped 48–72 h before surgery requiring general anesthesia, and during periods of low fluid intake. Ask your doctor before taking new medicines
*Use in the elderly* At ages over 60 years, doses and blood levels of lithium are at the low end of the therapeutic range (e.g., 0.4–0.6 mEq/L). Undesirable effects in the elderly in addition to those already described can include: confusion or worsening of cognitive functions, unsteady balance (ataxia), restless movements (akathisia), declining kidney function, hypothyroidism, possible worsening of diabetes, and leg-swelling (peripheral edema)
*Pregnancy* Lithium is used cautiously in pregnancy, with at least monthly monitoring of blood concentrations. If possible, lithium should be discontinued slowly or the dose lowered during the first trimester because of the association between lithium use and congenital malformations (birth defects) in this early period. Discuss risks and benefits of continuing, lowering, or interrupting lithium treatment during pregnancy and after childbirth with your doctor. During the third trimester, lithium blood levels should be monitored weekly. It is not necessary to stop lithium before delivery
*Postpartum* If lithium is discontinued during pregnancy, it should be restarted immediately after delivery, due to increased risk of relapses then. Target lithium levels should be relatively high (0.8–1.0 mEq/L) temporarily during the first month after delivery to minimize relapse risk, and checked twice weekly during the first 2 weeks after delivery. Do not breast-feed while taking lithium
*Lithium intoxication* Signs of high lithium blood levels include: severe tremor, confusion, vomiting, abdominal pain, diarrhea, speech difficulties, cardiac arrhythmia, hypotension, and convulsions. Some signs of intoxication may occur despite normal plasma levels of lithium. If the blood level of lithium is above 2 mEq/L, dialysis may be needed to remove lithium. Call your doctor or an emergency number immediately on suspicion of lithium intoxication
*Discontinuation* Interrupting lithium should be done gradually and under medical supervision to avoid relapse into mania or depression, unless an acute medical problem requires rapid discontinuation under close medical supervision. Dose-reduction of lithium can be done safely by lowering the daily dose by 20–25% every 2 weeks
